# Seroprevalence and risk factors of brucellosis in Arabian horses

**DOI:** 10.1002/vms3.759

**Published:** 2022-02-05

**Authors:** Zahra Lotfi, Mahdi Pourmahdi Borujeni, Masoud Ghorbanpoor, Ali Reza Ghadrdan Mashhadi

**Affiliations:** ^1^ Department of Food Hygiene Faculty of Veterinary Medicine Shahid Chamran University of Ahvaz Ahvaz Iran; ^2^ Department of Pathobiology Faculty of Veterinary Medicine Shahid Chamran University of Ahvaz Ahvaz Iran; ^3^ Department of Clinical Sciences Faculty of Veterinary Medicine Shahid Chamran University of Ahvaz Ahvaz Iran

**Keywords:** brucellosis, diagnosis, epidemiology, horse, zoonotic disease

## Abstract

**Background:**

Brucellosis, as a zoonotic disease, mainly occurs in horses by *Brucella abortus, Brucella canis* and *Brucella suis*. The disease in equines is often asymptomatic, but the clinical signs in horses are mostly characterized by bursitis, arthritis and tenosynovitis.

**Objectives:**

This study, thus, aimed to determine the seroprevalence of brucellosis and its associated risk factors in the Arabian horses of Khuzestan province, South‐west Iran.

**Methods:**

To that end, the blood samples randomly collected from 180 Arabian horses were analyzed for the presence of anti‐*Brucella* antibodies by Rose Bengal plate test (RBPT), serum agglutination test (SAT), 2‐mercaptoethanol test (2‐ME) and a commercial i‐ELISA kit.

**Results:**

The ROC curve analysis showed that the best cut‐off point for *S/P* values in i‐ELISA turned out to be 26.25%. The results showed that the overall seroprevalence of brucellosis based on parallel interpretation of the test results was 12.22% (Positive/Tested = 22/180). The prevalence of acute and chronic brucellosis was 8.3 and 3.9%, respectively. The seroprevalence of brucellosis with RBPT and i‐ELISA methods was 1.11% (2/180) and 7.22% (13/180), respectively. According to what SAT revealed, 9.44% (17/180) of sera had a titer of 40 or greater, and at 2‐ME, 7.22% of samples (13 out of 180 samples) depicted a titer of 40. The results of i‐ELISA, SAT and 2‐ME were significantly different from those of RBPT (*p *< 0.01); however, there was no significant difference between i‐ELISA, SAT and 2‐ME in findings (*p *> 0.05).

**Conclusions:**

The results of this study recommend that i‐ELISA be used for screening purposes of brucellosis in horses. The findings confirmed that Arabian horses are natural hosts for the Brucellae. It is, thus, necessary to adopt appropriate prevention and control programs by health authorities and horse owners so as to reduce the distribution and transmission of the infection in the regions where brucellosis is prevalent.

## Graphical abstract

In the present study, in addition to determining the prevalence of brucellosis infection in the Arabian horses in the southwest of Iran through serological methods including Rose Bengal plate test, serum agglutination test, 2‐mercaptoethanol test, and i‐ELISA test, the serological methods used were also evaluated and the associated risk factors affecting the infection were identified, as well. The results showed that the sensitivity of i‐ELISA was greater than RBPT, so it is recommended that i‐ELISA be used for screening purposes of brucellosis in Arabian horse population due to its short time required to perform, relatively simple and standardized implementation, measuring a large number of samples simultaneously and cost‐effectiveness. The findings confirmed that Arabian horses are natural hosts for the Brucellae.

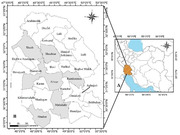

## 1 INTRODUCTION

Brucellosis is one of the most important zoonotic diseases with a global distribution that affects many humans and animals (Corbel, 1997). Equine brucellosis is caused by *Brucella abortus*, *Brucella suis* and *Brucella canis* and the natural infection may be caused through ingestion of infected material, respiratory system or skin wounds (Lucero et al., 2008). Many horses enter a latent infection state following infection, and despite having positive agglutination titer, they do not show clinical symptoms. However, non‐specific symptoms such as weakness, depression, muscle stiffness, intermittent fever and movement disorders are seen in some horses infected with *Brucella*. Furthermore, brucellosis in equines may be associated with fistulous withers, inflammation of the atlantal bursa, carpal bursitis, tenosynovitis, osteomyelitis, osteoarthritis and rarely reproductive disorders (Cohn et al., 1992; Cvetnic et al., 2005; Ocholi et al., 2004). Despite the high cost of controlling brucellosis, as an endemic disease in Iran, through vaccination, testing and slaughtering of domestic ruminants, this disease has been seen in all parts of the country for more than half a century, and thousands of people are infected each year, as well. The average incidence of brucellosis in the Iranian human population was 21 cases per 100,000 populations, although this varied between 1.5 and 107.5 per 100,000 population in different parts of the country (Zeinali et al., 2011). Also, the serological prevalence of equine brucellosis in some parts of Iran varied from 0 to 12% (Badiei et al., 2013; Gharekhani et al., 2020; Ghobadi & Salehi, 2013; Hashemitabar & Poursafar, 2005; Nemati, 2017; Rafeiei Sharebabaki, 2018; Tahamtan et al., 2008, 2010; Taheri, 2018). The prevalence of brucellosis infection in horses depends on factors such as the status of management and health measures, host determinants, sample size, and diagnostic methods used. Culture and serology are mainly used to detect the equine infection. However, as it is difficult to cultivate and isolate Brucellae, serological methods including Rose Bengal plate test (RBPT), serum agglutination test (SAT), 2‐mercaptoethanol test (2‐ME), milk ring test, indirect and competitive ELISA (i‐ELISA and c‐ELISA), fluorescence polarization assay, and complement fixation test are preferred (Antunes et al., 2013; Ducrotoy et al., 2018; Esmaeili, 2014; Hussain et al., 2020; Tel et al., 2011). However, among these serological methods, the SAT is able to detect the total amount of agglutinin antibodies, including IgM and IgG (mainly IgM), whereas 2‐ME and i‐ELISA measure IgG antibodies (Godfroid, et al., 2010; Roushan et al., 2010).

Considering the traditional to semi‐industrial livestock production and breeding systems in the southwest Iran, horses, cattle, buffaloes, sheep and goats are usually kept together, and the transmission of pathogens between them is thus likely. A review of the literature shows that due to the importance of brucellosis in humans and domestic ruminants, most epidemiological studies conducted so far in Iran have mainly focused on determining the seroprevalence of brucellosis and its associated risk factors in these species, paying less attention to horses. In addition, no control programs, including vaccination, testing and slaughter of positive cases, unlike cattle, sheep and goats, are performed for horses, donkeys and mules in Iran (Esmaeili, 2014). Thus, the epidemiological study of brucellosis in the Arabian horses is important because in addition to its pathogenicity for horses, it can be transmitted from horses to humans and other animals. Certainly, to prevent and, in particular, eradicate a disease, its agent hosts should be considered. Brucellae are no exception to the rule. Therefore, in the present study, in addition to determining the prevalence of brucellosis infection in the Arabian horses in the southwest of Iran through serological methods, including RBPT, SAT, 2‐ME and i‐ELISA, the serological methods used were also evaluated and the associated risk factors affecting the infection were identified, as well.

## 2 MATERIALS AND METHODS

### 2.1 Study area and population

**FIGURE 1:**
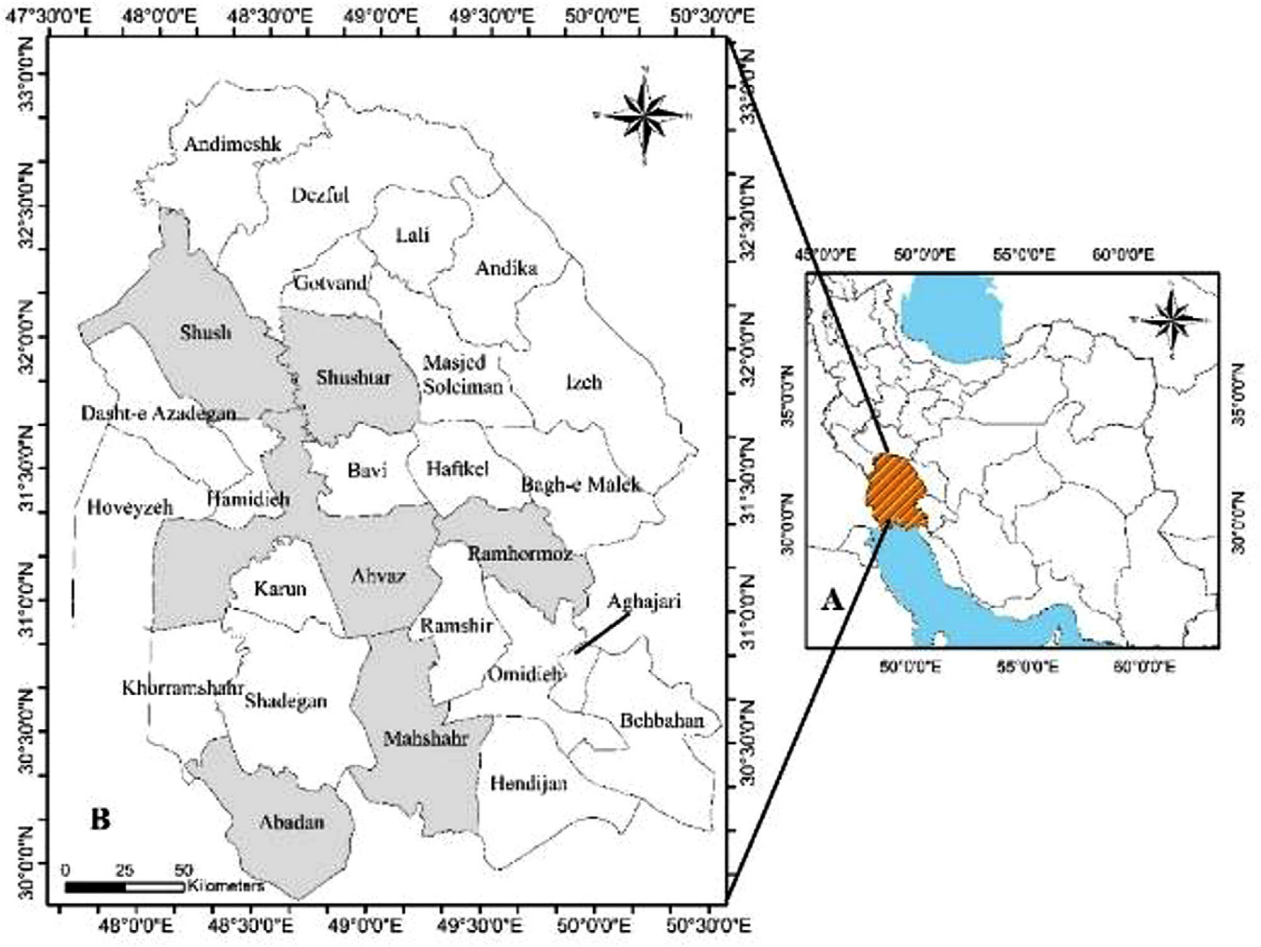
Geographical location of Khuzestan Province in the southwest of Iran

This epidemiological study was carried out in Khuzestan Province located in the southwest of Iran (Figure 1). The topographic elevations of this tropical province, located between 48°E and 49.5°E longitudes and 31°N and 32°N latitudes, with an area of 63,213 km^2^ and 27 cities varies between 0 and 3740 m. The climate of Khuzestan Province varies from arid to humid and its northern parts experience cold weather, whereas its southern parts experience tropical weather (Zarasvandi et al., 2011). Therefore, to create regional differences in the epidemiological determinants, such as environment and management, Khuzestan province was divided into four different regions from which, one or two cities were randomly selected. Khuzestan province is one of the important breeding areas for Arabian horses in the southwest of Iran where a significant population of such horses (about 3500 horses) is kept and bred, mainly for racing and breeding. Certainly, this livestock has some direct and indirect effects on the economy of the region's residents, it is thus, important for health policymakers, veterinarians and horse owners to take major steps to determine the prevalence of equine diseases, especially zoonotic diseases in this particular breed. Also, more than 300,000 cattle, 3.5 million sheep and 2.1 million goats are kept in Khuzestan Province (Census, 2016; Statistical Center of Iran, 2017).

### 2.2 Sample and data collection

To investigate the seroprevalence of brucellosis in the Arabian horses of Khuzestan Province, 180 blood samples (10 mL from the jugular vein by venoject [EXPILAB, Gel & Clot Activator] from each animal) were taken according to two‐stage random sampling method from the horses of six cities of the province, including Abadan, Ahvaz, Mahshahr, Ramhormoz, Shoush and Shoushtar. After serum separation, the samples were stored at −20°C until use. The characteristics of horses, including age (year), sex (male or female), history of leaving the province (yes or no), body condition score (good including a moderate to good body condition score, or bad including an emaciated to poor body condition score), type of use (racing, breeding or mix of both), herd size (number of horses) and geographical location (Abadan, Ahvaz, Mahshahr, Ramhormoz, Shoush or Shoushtar) were recorded simultaneously with blood sampling, as well. In addition to, none of the horses had any history of clinical signs of brucellosis at the time of blood collection.

### 2.3 Serological analysis

All 180 collected serum samples were evaluated for anti‐*Brucella* antibodies by RBPT, SAT (Wright test) and 2‐ME using whole cell antigen (Razi Vaccine and Serum Research Institute, Iran) according to OIE manual, and a commercial indirect IgG ELISA test (ID vet, France, ID Screen Brucellosis Serum Indirect Multi‐species). The SAT was considered positive when titer was at least 40 (Alton et al., 1988; Denny, 1972; Tel et al., 2011; Yilmaz & Wilson, 2013). The ID vet ELISA kit was initially designed to diagnose brucellosis in cattle, sheep, goats and pigs, and there was no information on the possibility of using it for the diagnosis of horse brucellosis. Therefore, to use i‐ELISA kit in the present study, its efficiency for the diagnosis of brucellosis in horses was initially analyzed by several equine sero‐positive and sero‐negative samples. Having confirmed that i‐ELISA kit was also suitable for the detection of anti‐*Brucella* antibodies in horse serum samples, we calculated its appropriate cut‐off for diagnosis of equine brucellosis. For this purpose, 90 equine sera (70 Wright‐sero‐negative and 20 sero‐positive) were assessed according to the kit manufacturer's instructions. The optical density of samples (OD_Sample_), kits positive (OD_PC_) and negative controls (OD_NC_) were recorded and the *S/P*% was calculated for each sample according to the following formula:

$$S/P\%=\frac{OD_{\mathrm{Sample}}-OD_{\mathrm{NC}}}{OD_{\mathrm{PC}}-OD_{\mathrm{NC}}}\times \;100$$

The cut‐off point of the i‐ELISA kit for the diagnosis of equine brucellosis was calculated by ROC (receiver operating characteristic) curve analysis.

### 2.4 Statistical analysis

Statistical analysis of data was performed using SPSS (version 22.0; SPSS for Windows Inc., Chicago, Illinois). The association between age (1–2 years, 3–9 years or ≥ 10 years), sex (male or female), history of leaving the region (yes or no), body condition score (good or bad), type of use (racing, breeding or racing and breeding), herd size (1‐4 horses, 5–9 horses or ≥ 10 horses) and geographical location (Ahvaz, Abadan, Mahshahr, Ramhormoz, Shoush or Shoushtar) were analyzed by the Chi‐square test and univariable and multivariable logistic regression (calculation of odds ratio) in horse level. Risk factors associated with brucellusis (*p* ≤ 0.25) in univariable logistic regression were further analyzed in a multivariable logistic regression model, using a backward, step‐wise algorithm. The goodness of fit of the model was determined using the Hosmer and Lemeshow test. Comparison of diagnostic methods was performed with Cochran and McNemar tests and kappa statistic (prevalence‐ and bias‐adjusted Kappa statistic). Also, estimation of confidence intervals for prevalence was calculated by the Agresi–Coull method (Thrusfield et al., 2018). Differences were considered statistically significant (*p *≤  0.05). The map was drawn using ArcGIS software version 10.3.

## 3 RESULTS

### 3.1 Seroprevalence of *Brucella*

**FIGURE 2:**
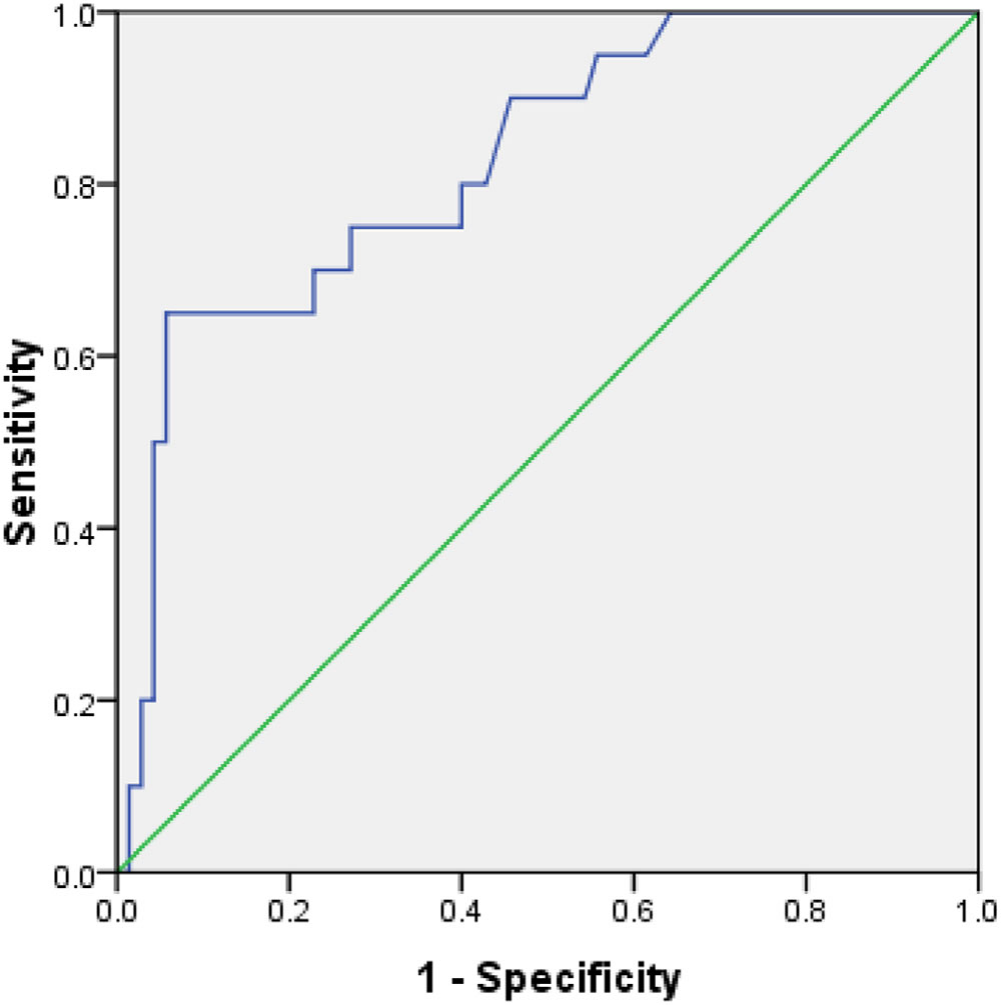
ROC curve for i‐ELISA for diagnosing *Brucella* infection in the Arabian horses

**FIGURE 3:**
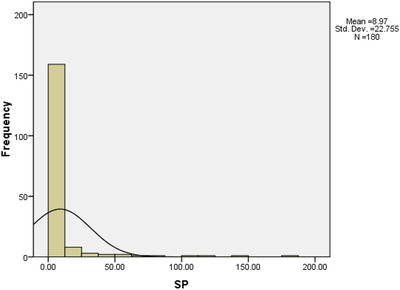
Absolute frequency of *S/P* values for brucellosis in the Arabian horses in the southwest of Iran

The overall seroprevalence of brucellosis (acute and chronic) was 12.22% (22 samples out of 180 samples, 95CI: 8.16–17.84%). The prevalence of acute and chronic brucellosis was 8.3% (15 out of 180 samples) and 3.9% (7 out of 180 samples), respectively (Table 1). The seroprevalence by the RBPT was found to be 1.11% (2 samples out of 180 samples, 95CI: 0.3–3.95%). Considering the cut‐off point of 40 and greater, the seroprevalence of brucellosis by the SAT was, in turn, 9.44% (17 samples out of 180 samples, 95CI: 5.14–13.74%). The analysis of ROC curve unveiled that the best cut‐off point for *S/P* values in the ID vet ELISA (sensitivity = 65% and specificity = 94.3%) was 26.25% (the area under the curve [AUC] = 0.83, 95CI: 0.73–0.93, *p *< 0.001) (Figure 2). The seroprevalence of brucellosis by the i‐ELISA test was 7.22% (13 samples out of 180 samples, 95CI: 3.44–11%). The frequency distribution of *S/P* values is presented in Figure 3. At 2‐ME, 7.22% of samples (13 out of 180 samples, 95CI: 3.44–11%) depicted a titer of 40.

**TABLE 1:** Absolute frequency of positive samples (+) based on Rose Bengal plate test (RBPT), serum agglutination test (SAT), 2‐mercaptoethanol test (2‐ME) and i‐ELISA

Diagnostic test Frequency	RBPT	SAT	2‐ME	i‐ELISA	Brucellosis
2	+	–	–	–	Inconclusive
2	–	+	‐	+	Acute
4	–	+	+	+	Acute
9	–	+	+	–	Acute
2	–	+	–	–	Inconclusive
7	–	–	–	+	Chronic

### 3.2 Evaluation of serological methods

The Cochran's Q test showed that there was no significant difference between diagnostic methods including RBPT, SAT, 2‐ME and i‐ELISA (Cochran's Q = 16.82, df = 3, *p *= 0.001). A comparison of the 2‐ME and SAT tests showed that all negative cases in SAT were negative in 2‐ME and also many positive cases in the SAT were also positive in 2‐ME (Table 2). Comparison of the 2‐ME and i‐ELISA showed that many positive cases in 2‐ME were negative in i‐ELISA and also many positive cases in i‐ELISA were negative in 2‐ME, but many negative cases in the 2‐ME were also negative in i‐ELISA (Table 3). Statistical analysis showed that the 2‐ME did not differ significantly from the ID vet ELISA and SAT in the diagnosis of equine brucellosis (*p *> 0.05). Comparison of the i‐ELISA and SAT showed that many positive cases in SAT were negative in i‐ELISA and also some positive cases in i‐ELISA were negative in SAT (Table 4). The McNemar test showed that the SAT did not differ significantly from the ID vet ELISA in the diagnosis of equine brucellosis (*p *> 0.05).

**TABLE 2:** Comparison of 2‐mercaptoethanol test (2‐ME), serum agglutination test (SAT) in the diagnosis of brucellosis in the Arabian horses

SAT 2‐ME	Positive	Negative	Total
Positive	13	0	13
Negative	4	163	167
Total	17	163	180

Accuracy = ([13 + 163]/180) × 100 = 97.78%, Kappa statistic = 0.86 (almost perfect agreement based on Dohoo et al., 2003), PABAK (prevalence‐and bias‐adjusted kappa statistic) = 0.96 (*p *< 0.001).

**TABLE 3:** Comparison of i‐ELISA and 2‐mercaptoethanol test (2‐ME) in the diagnosis of brucellosis in the Arabian horses

i‐ELISA 2‐ME	Positive	Negative	Total
Positive	4	9	13
Negative	9	158	167
Total	13	167	180

Accuracy = ([4 + 158]/180) × 100 = 90%, Kappa statistic = 0.25 (fair agreement based on Dohoo et al., 2003), PABAK = 0.8 (*p *< 0.001).

**TABLE 4:** Comparison of i‐ELISA and serum agglutination test (SAT) in the diagnosis of brucellosis in the Arabian horses

i‐ELISA SAT	Positive	Negative	Total
Positive	6	11	17
Negative	7	156	163
Total	13	167	180

Accuracy = ([6 + 156]/180) × 100 = 90%, Kappa statistic = 0.35 (fair agreement based on Dohoo et al., 2003), PABAK = 0.8 (*p *< 0.001).

Comparison of the SAT and 2‐ME with RBPT revealed that all positive cases in the SAT or 2‐ME were negative in the RBPT. Besides, it was found that all cases that were positive in the RBPT were not detectable in the SAT or 2‐ME, but many negative cases in the SAT or 2‐ME were also negative in RBPT. The results of RBPT were significantly different from the SAT (*p* < 0.001) and 2‐ME (*p *= 0.007) in the diagnosis of equine brucellosis. Generally, there was a significant difference between the two methods (*p *= 0.001). A comparison of the RBPT and i‐ELISA tests showed that all positive cases in RBPT were negative in ID vet ELISA, and all positive cases in ID vet ELISA were negative in RBPT. Rose Bengal was significantly different from the ID vet ELISA (*p *= 0.007).

### 3.3 The role of associated factors

To investigate the risk factors affecting the seroprevalence of brucellosis at the horse level, the overall seroprevalence of brucellosis based on parallel interpretation of the test results was used and the strength of association of them is summerized in Table 5. Examination of variables related to *Brucella* infection in univariable logistic regression, including sex and geographical location (*p *≤ 0.25), with multivariable logistic regression showed that none of them (*p *= 0.11 for sex and *p *= 0.29 for geographical location) had a significant effect on the infection.

**TABLE 5:** Univariable association of demographic and other variables with brucellosis in the Arabian horses of Iran

Variable	Category	Prevalence (Positive N./Total N.)	Odds Ratio (OR)	95% CI for OR	*p*‐Value
**Age**	1–2 years	10.71% (6/56)	1	–	–
	3–9 years	12.77% (12/94)	1.22	0.43–3.45	0.71
	≥10 years	13.33% (4/30)	1.28	0.33–4.95	0.72
**Sex**	Male	7.58% (5/66)	1	–	–
	Female	14.91% (17/114)	2.14	0.75–6.09	0.16
**Body condition**	Good	11.69% (20/171)	1	–	–
	Bad	22.22% (2/9)	2.16	0.42–11.11	0.36
**Herd size**	1‐4 horses	16.67% (6/36)	1.65	0.42–6.42	0.47
	5–9 horses	10.81% (4/37)	1	–	–
	≥10 horses	11.21% (12/107)	1.04	0.31–3.46	0.95
**History of leaving the province**	No	12.15% (13/107)	1	–	–
	Yes	12.32% (9/73)	1.02	0.41–2.52	0.97
**Type of use**	Racing and breeding	9.86% (7/71)	1	–	–
	Racing	15.69% (8/51)	1.7	0.57–5.04	0.34
	Breeding	12.07% (7/58)	1.26	0.41–3.81	0.69
**Geographical location**	Abadan	0% (0/14)	–	–	–
	Ramhormoz	5% (1/20)	1	–	–
	Shoushtar	8.57% (3/35)	1.78	0.17–18.37	0.63
	Shoush	8.57% (3/35)	1.78	0.17–18.37	0.63
	Mahshahr	14.29% (3/21)	3.17	0.3–33.31	0.34
	Ahvaz	21.82% (12/55)	5.3	0.64–43.75	0.12

## 4 DISCUSSION

In this epidemiological study, the seroprevalence of brucellosis was determined in Arabian horses in south‐western Iran (Khuzestan Province). Brucellosis is a worldwide zoonosis posing heavy economic and public health damage. The results of this study could be a reflection of brucellosis seroprevalence in other livestock and humans in the region, and would determine the role of Arabian horses in disease epidemiology. The findings can also help health policymakers make better decisions to control and prevent the disease.

As the findings showed, the seroprevalence of brucellosis reported in this study using RBPT (1.11%), SAT (9.44%), 2‐ME (7.22%) and ELISA test (7.22%) was in the range of the proportion (0–10%) previously reported in Iran (Badiei et al., 2013; Gharekhani et al., 2020; Ghobadi & Salehi, 2013; Hashemitabar & Poursafar, 2005; Nemati, 2017; Rafeiei Sharebabaki, 2018; Tahamtan et al., 2008; Tahamtan et al., 2010; Taheri, 2018). Therefore, despite differences in timing, environment and host determinants, diagnostic tests, sampling size and study designs, the prevalence of brucellosis in the Iranian horses is almost the same, and the seroprevalence of brucellosis is expected to be less than 10% across Iran. In the studies conducted in other countries, the seroprevalence of brucellosis were reported to be 0—100% in Nigeria (Ardo & Abubakar, 2016; Ardo et al., 2016; Bertu et al., 2014; Ehizibolo et al., 2011; Sadiq et al., 2013), 8.3% in Mongolia (Zolzaya et al., 2014), 0.26–6.5% in Brazil (Antunes et al., 2013; Santos et al., 2016), 3.6‐ ‐67.9% in Pakistan (Gul et al., 2013; Hussain et al., 2020; Safirullah et al., 2014), 0.25–60.6% in Turkey (Göz et al., 2007; Solmaz et al., 2004; Tel et al., 2011), 0.24% in Mexico (Acosta‐Gonzalez et al., 2006), 3.6–4.9% in Sudan (Musa, 2004), 1–8.5% in Jordan (Abo‐Shehada et al., 2009) and 0% in Eritrea (Omer et al., 2000). This significant difference in the prevalence of brucellosis in different countries, and even between different regions in a country can be due to differences in management such as husbandry, contact rate with domestic and wild animals and population density, location, climate, sample size, diagnostic method and hosting characteristics (Acosta‐Gonzalez et al. 2006; Gul & Khan, 2007). However, some of these differences may also be due to differences in the minimum ratio of the disease in agglutination tests in different countries and regions (Pinset & Fuller, 1997; Reed & Bayly, 1998; Wintzer, 1986).

Serological techniques can pinpoint the potent humoral immune responses, triggered by exposure to *Brucella*. In such cases, the emergence of IgG antibodies occurs far later than IgM. Such antibodies do not disappear even after the response reaches its highest point (3‐4 weeks post‐infection) and thus, can be identified over longer duration of time (up to several years); quite conversely, despite the swift induction of IgM antibodies (2–3 weeks after exposure), they linger merely for a few months (Godfroid et al., 2002; Saegerman et al., 2004; Sutherland, 1984). What differentiates acute infections from chronic ones is the kinetics of production, the emergence of the principal immunoglobulin isotypes over the course of the infection, and the function of these immunoglobulins in various serological trials. Although the simultaneous presence of IgM (detected in an agglutination test) and IgG (detected in i‐ELISA and 2‐ME) signifies acute brucellosis, the sole presence of IgG characterizes the chronic brucellosis (Godfroid et al., 2010). In this study, the prevalence of acute brucellosis was relatively higher than the chronic form (8.3 vs. 3.9%). However, the horses featured no clinical signs, such as weakness, depression, muscle stiffness, intermittent fever, fistulous withers, reproductive disorders and movement disorders. Under multi‐species housing in Khuzestan province, horses are grazed, watered and kept in a close contact with cattle, sheep and goats. Therefore, horses usually become infected through ingesting the *Brucella*‐contaminated feed and water, and they would show the sub‐clinical and asymptomatic forms of the infection.

There was also a significant difference between the serological diagnostic methods used in terms of findings. As such, all of the positive cases in RBPT were negative in SAT, 2‐ME and i‐ELISA tests, possibly due to non‐specific reactions in RBPT (Young, 1991). In addition, many of the positive cases in the SAT were negative in i‐ELISA, indicating that there were some non‐specific reactions in SAT as compared to i‐ELISA. In the RBPT and Wright test (SAT), the whole *Brucella abortus* bacterin is used as an antigen, some of which are similar to those of other pathogens. For example, lipopolysaccharide O‐antigen of *Brucella* has molecular mimicry with *Salmonella*, *Escherichia coli* O116 and O157, *Pseudomonas maltophilia* and *Yersinia enterocolitica* O:9 (Kuila et al., 2017; Yohannes et al., 2012). On the other hand, in the RBPT and SAT, only anti‐surface antigen antibodies are detected, while in the ELISA kit, specific antigens are used, and therefore, the results are more specific. Nevertheless, although IgG tracking is the underpinning principle of the design of ELISA commercial kits, various sub‐classes and allotypes of IgG antibodies might appear in animals. For instance, seven IgG sub‐classes with differing operational and structural features exist in horses (Lewis, et al., 2008). Consequently, false‐negative results may arise if these commercial kits are deployed for studying infectious diseases in certain animals. In this study, a number of samples turned out negative for i‐ELISA test despite demonstrating the IgG titer in Wright and 2‐ME‐Wright tests. In this research, no statistically significant differences were defined between the SAT, 2‐ME and i‐ELISA results; this issue indicated the higher level of the IgG than IgM in the tested serums. However, the non‐significant difference between of the mentioned tests, suggested existence of dissimilar percent of the specific anti‐*brucella* IgG sub‐classes in the infected animals. IgG sub‐classes do not have the same tendency to agglutinate the bacteria or bind to the conjugated anti‐globulin antibodies.

Ardo and Abubakar (2016) reported that the prevalence of *Brucella* infection in horses was higher in the RBPT than in the Wright test, which contradicted the findings of the present study. The difference between the antigens used in these two methods is only in their pH and the coloured antigen used in RBPT. Accordingly, the animal needs to have a minimum titter of 40 in the Wright test to be positive in RBPT. On the other hand, if brucellosis is acute and IgM antibodies are produced, a stronger reaction is observed in the RBPT than in cases where the disease is chronic and there are more IgG antibodies in the serum. IgM antibodies is indeed stronger agglutinin than IgG (Racine & Winslow, 2009). Since the horses studied in this study did not show signs of brucellosis during sampling, it appears that the positive cases are related to early stages of disease, in which case there is high expectation of high titer of antibodies against *Brucella*.

Given the short time required to perform the test, its use for a variety of antigens, and its ability to measure a large number of samples simultaneously, as well as its lower error rate and cost‐effectiveness, ELISA, as the most accurate method of serological diagnosis of brucellosis, can thus, replace other serological tests already used for the diagnosis of brucellosis in horses. Moreover, since the control of brucellosis in European countries is done through a hygienic method, that is, not using the vaccine but eliminating the positive cases in the test, the ELISA kits that are designed and marketed in these countries are very sensitive considering their low cut‐off point so that they can track low antibody levels, and also detect and eliminate positive and even suspicious cases in the test. However, the control of brucellosis in Iran is done largely by vaccination, tests and slaughtering the positive cases. Therefore, the use of kits made in European countries is not recommended and it is suggested that their cut‐off points be recalculated and localized before use, as it was done in the present study. As such, the best cut‐off point for the kits used was determined by the ROC curve.

Although the presence of anti‐*Brucella* antibodies disclosed the exposure to *Brucella* spp., the exact *Brucella* species responsible for inducing the secretion of those antibodies remains unclear due to the limitations the current study faced with. In addition, due to the vulnerability of nearly all animal species to *Brucella* infection and consequently, the loss of antibody titres, there exists a high likelihood that the actual prevalence of brucellosis is shown higher than that unveiled by antibody detection. It is noteworthy that a positive result yielded by the agglutination test, chiefly utilized for the identification of IgM, cannot confirm brucellosis if it is not substantiated by i‐ELISA within 1 week, an obstacle the present cross sectional study (parallel testing) failed to overcome (Godfroid, et al., 2010).

## 5 CONCLUSIONS

This epidemiological study showed that the *Brucella* infection is relatively prevalent (about 10%) in Arabian horses in the southwest of Iran and confirmed that they are the natural hosts of the Brucellae, although the infection is latent or sub‐clinical in them. Even though, the role of horses in transmitting *Brucella* to other animals is less important, they can play a significant role in the epidemiology of the disease as a reservoir or secondary host to preserve this bacterium. The results showed that the sensitivity of i‐ELISA was more than RBPT, so it is recommended that i‐ELISA be used for screening purposes of brucellosis in Arabian horse population due to its short time required to perform, relatively simple and standardized implementation, measuring a large number of samples simultaneously and cost‐effectiveness. Accordingly, the role of horse as an intervening factor in the control of brucellosis in human should be carefully considered by healthcare providers. Given the limited number of literature in this field, the findings of the present study can be helpful in conducting other epidemiological studies and controlling programs.

## CONFLICT OF INTEREST

The authors of this manuscript declare no conflict of interest.

## ETHICS STATEMENT

This study was “observational study” and the research protocol was reviewed and approved by research committee of the Faculty of Veterinary Medicine, Shahid Chamran University of Ahvaz and documented by the number: 96791135. Before beginning work on the present study, we contacted the horse owners and written informed consent was obtained from the owners for the participation of their animals in this study. During collecting blood specimens, these animals were not disturbed.

## AUTHOR CONTRIBUTIONS

Zahra Lotfi: Data curation (equal); Investigation (equal); Methodology (equal); Validation (equal); Visualization (equal); Writing – review & editing (equal). Mahdi Pourmahdi Borujeni: Conceptualization (lead); Data curation (equal); Formal analysis (lead); Investigation (equal); Methodology (equal); Project administration (lead); Resources (equal); Software (lead); Supervision (lead); Validation (equal); Visualization (equal); Writing – original draft preparation (lead); Writing – review & editing (equal). Masoud Ghorbanpoor: Data curation (equal); Investigation (equal); Methodology (equal); Resources (equal); Validation (equal); Visualization (equal); Writing – review & editing (equal). Ali Reza Ghadrdan Mashhadi: Data curation (equal); Investigation (equal); Methodology (equal); Resources (equal); Writing – review & editing (equal).

### PEER REVIEW

The peer review history for this article is available at https://publons.com/publon/10.1002/vms3.759

## Data Availability

The data that support the findings of this study are available from the corresponding author upon reasonable request.
